# Efficient Alpha Radiation Detector using Low Temperature Hydrothermally Grown ZnO:Ga Nanorod Scintillator

**DOI:** 10.1038/s41598-019-47732-1

**Published:** 2019-08-06

**Authors:** R. M. Sahani, Chandni Kumari, Arun Pandya, Ambesh Dixit

**Affiliations:** 1 Nuclear Radiation Management and Application Division, Defence Laboratory (DRDO), Jodhpur, 342011 India; 2 Department of Physics, Indian Institute of Technology Jodhpur, Jodhpur, 342037 India

**Keywords:** Sensors and biosensors, Characterization and analytical techniques

## Abstract

We report efficient detection of alpha radiation on highly textured and vertically aligned along (002) Gallium-doped Zinc Oxide (ZnO:Ga) nanorods on a glass substrate with an average diameter ~150 ± 10 nm. Photoluminescence measurement showed near band emission 393 nm, in agreement with the bandgap value ~3.22 eV, measured by UV-Vis spectroscopy. The developed ZnO:Ga nanorod scintillator is coupled with a commercially available photomultiplier tube and 1K Multichannel Analyser to fabricate an alpha radiation detector. The performance of the alpha radiation detector is evaluated using various activities alpha radiation sources. A large pulse height spectrum is recorded by the detector for different alpha sources against the background spectrum. The calculated detection efficiency and Minimum Detectable Activity (MDA) showed that the detector is highly sensitive to alpha radiation. The repeatability and reproducibility of the performance are studied by evaluating the response of a single scintillator for numerous exposures and by studying inter-batch response variations, respectively. The response is repeatable within ±1% whereas reproducibility varies from ±20% for extremely low activity alpha sources to ±5% for high activity alpha sources. The performance of ZnO:Ga nanorod scintillator grown on glass substrate demonstrates that it can be a promising material system for the detection of alpha radiation.

## Introduction

Alpha radiation, one form of Ionizing radiation, is a stream of doubly charged particles and is emitted from radioactive decay of a heavy nucleus, D-T or D-D reactions^1^. Isotopes of uranium and plutonium are alpha emitters with their application in nuclear reactors and nuclear weapons as fissile material. In a neutron generator utilizing D-D to D-T reaction for emission of monoenergetic neutrons for nuclear material interrogation purpose, monitoring of alpha radiation generated in opposite to neutrons is used for associated particle imaging^2^. Due to high mass and charge, alpha radiation has a short range in the matter (41 mm in the air for 5.5 MeV), therefore external radiation hazard is minimum but in contrast, the internal radioactive contamination can cause the maximum radiation hazard^3^. Accidental release of radioactive material in the environment due to nuclear reactor accident or intentional explosion of nuclear weapon or radiological dispersal devices (RDDs) utilizing alpha emitters leads to radioactive contamination of the surrounding environment. The radioactive contamination in air, water, and food may reach to human internal organs due to their direct consumption or through the food chain or due to contact of broken/injured skin. There are possibilities of radioactive surface contamination at laboratories using alpha sources, nuclear processing facilities, and waste disposal facilities which must be restricted below a certain permissible level. Monitoring of alpha radiation is therefore required for emergency management and occupational radiation protection. Alpha radiation detection is also an important tool for associated particle imaging utilizing the generation 14.1 MeV neutron by D-T reaction in a neutron generator. The mapping of time and position of alpha particles produced in opposite to neutrons is a novel technique to ascertain the produced neutron direction and time. Thus, efficient alpha detectors having fast response are required for such applications.

Scintillation detectors such as polycrystalline silver doped zinc sulfide, ZnS(Ag), gadolinium pyrosilicate (GPS) and gadolinium aluminum gallium garnet (GAGG) single crystals are used for alpha radiation detection^4^. In the last two decades, work on zinc oxide in different forms such as phosphor, transparent ceramics, thin film, single crystal has been reported as a potential scintillator. Lehman *et al*. reported the fast response of the order of nanosecond for n-type ZnO^5,6^. gallium doping has served the robust and efficient n-type dopant in ZnO. The Ga doped ZnO (ZnO:Ga) phosphors are commonly synthesized by two different methods such as solid-state reaction of ZnO and Ga_2_O_3_, followed by H_2_ treatment and diffusion of Ga metal in ZnO. The former method resulted in fabricating ultrafast scintillator. These methods utilizing high temperature, vacuum and long processing time 10–15 hrs^7^. ZnO:Ga powder coated on optical fiber face plate has been used as an alpha detector in portable neutron generators^8^. Transparent zinc oxide ceramic is produced by hot pressing and spark plasma sintering of ZnO powder prepared by the different methods such as solution-phase, urea precipitation, and combustion processes. The hot pressing utilizes the high temperature and high pressure (around 1000 °C, 35 MPa)^9^. ZnO transparent ceramics prepared using uniaxial hot pressing resulted in large-sized grains^10^. ZnO and ZnO:Li transparent ceramics have shown their performance in detection of gamma and alpha radiations, respectively and comparable to that of CsI(Tl) detector^11^. ZnO:Zn transparent ceramics produced by hot pressing techniques shows X-ray induced luminescence, which is comparable to that of BGO^12^. Epitaxial ZnO:In thin films on ZnO single crystal substrate in different indium concentrations, have shown as alpha radiation detector^13^. Zinc oxide single crystals, grown by several methods such as high-pressure direct melting technique^14,15^, hydrothermal^16^, vapor phase method^17^, are used for detection of alpha radiation and pulsed X-ray detection. Alpha excited scintillations for ZnO, ZnO:In, Li, ZnO:Ga and ZnO:Er, Li single crystals are studied and responses are compared with standard plastic scintillator detector^18^. ZnO:In single crystal prepared using direct melting techniques exhibited the fastest response as compared to plastic scintillator^19^. Hydrothermally grown ZnO single crystal coupled with a position sensitive photomultiplier tube (PSPMT) is demonstrated as an alpha imager^20^ and ZnO:Sc single crystal as alpha radiation detector having sub-nanosecond decay time^21^.

Zinc Oxide nanorods are one-dimensional structure prepared using different techniques vapor-liquid-solid (VLS) method, metalorganic chemical vapor deposition (MOCVD) and hydrothermal method^22^ and shown potential in gas sensing, UV detection, and solar cells^23^. Hydrothermal method is the simplest technique used for ZnO nanorods which utilize seed formation followed by low-temperature solution growth. It is relatively simple, cost effective and energy saving process^24^ operating in 90–95 °C temperature window with respect to other synthesis techniques. Therefore, this process can be easily scaled for the growth of nanorods on large substrates whereas the other processes such as MOCVD, VLS etc. will require highly sophisticated instruments for the same. Synthesis of sputtering seeded hydrothermally grown Ga doped ZnO nanorods are investigated for their photoluminescence and alpha pulse height spectra^25^. In this paper we report the synthesis of highly textured and (002) oriented vertically aligned Ga doped ZnO nanorods utilizing spin coated seed layer. The synthesized nanorods on glass substrates are coupled to a photomultiplier tube and multichannel analyzer (MCA) to realize a compact alpha radiation detector. Alpha radiation response is investigated using different activities of alpha radiation sources. The repeatability of performance and reproducibility are ensured by using different nanorod scintillator samples.

## Results

### Structural and optical characterization

**Figure 1:**
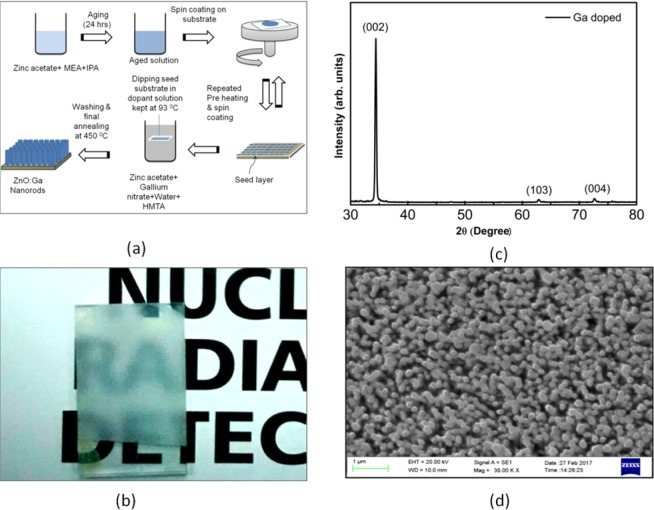
(**a**) Schematic of nanorod growth process, (**b**) grown nanorods (ZnO:Ga/Glass), showing transparent nature, (**c**) X-ray diffraction (XRD) pattern of naonorods, and (**d**) surface micrograph of grown ZnO:Ga nanorods.

Alpha radiation detector consists of (i) Ga doped ZnO nanorods, grown on the florine doped tin oxide (FTO) glass substrate, (ii) a photomultiplier tube, and (iii) a multichannel anlyzer. The synthesis of Ga doped ZnO nanorods consists of two steps (i) creation of ZnO seed layer on the substrate, and (ii) growth of Ga doped ZnO nanorods over the seeds. The seeding was done by depositing a seed layer by spin coating a gel consisting of an aged solution of zinc acetate dissolved in IPA with MEA. The coated layer is preheated in order to disassociate zinc acetate and form ZnO nano seeds. The repeated coating and preheating resulted in the desired seed layer. This seed layer is finally heated at higher temperature of ~300 °C for crystallizing ZnO seeds. These seeded substrates are kept in an aqueous solution containing gallium and zinc precursors in Hexamethylenetetramine (HMTA). The complete synthesis steps are summarized in Fig. 1(a), showing Ga doped ZnO nanorods on the seeded substrate, which are finally heated at 450 °C. These nanorods exhibit highly unidirectional growth along c axis (002) (Fig. 1c). The optical image of ZnO:Ga/Glass, Fig. 1b, support the semitransparency of these nanorods and SEM micrograph, Fig. 1d, confirms the uniform distribution of nanorods on the entire substrate. The diameter of these nanorods is about 150 ± 10 nm.

**Figure 2:**
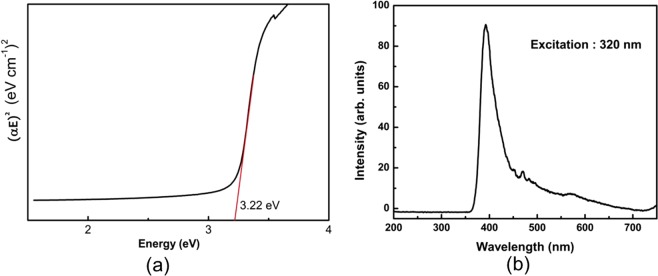
(**a**) (α.E)^2^ versus energy spectrum for ZnO:Ga nanorods with red line showing the extrapolation for zero absorption (X-axis intercept value is marked, representing the optical band gap) and (**b**) Emission spectra of ZnO:Ga nanorods with UV excitation on 320 nm.

The optical absorption, α, is converted (α.E)^2^ and plotted against energy, E, Fig. 2a and a tangential linear region exhibiting higher absorption is extrapolated and the intercept on X-axis is considered as the optical bandgap for the system. The noticed bandgap value is about 3.22 eV, which slightly less than the band gap 3.37 eV of pristine ZnO nanorods. The reduction in band gap is attributed to the Ga doping in ZnO, where Ga defect states may lie below the ZnO conduction band, causing the observed lowering in the absorption edge. The photoluminescence (PL) spectrum is shown in Fig. 2b using 320 nm excitation, exhibiting a sharp peak at 393 nm which is near band emission and is consistent with observed band gap value from the optical absorption measurements.

## Alpha radiation response

### Response to the different activity of alpha sources

**Figure 3:**
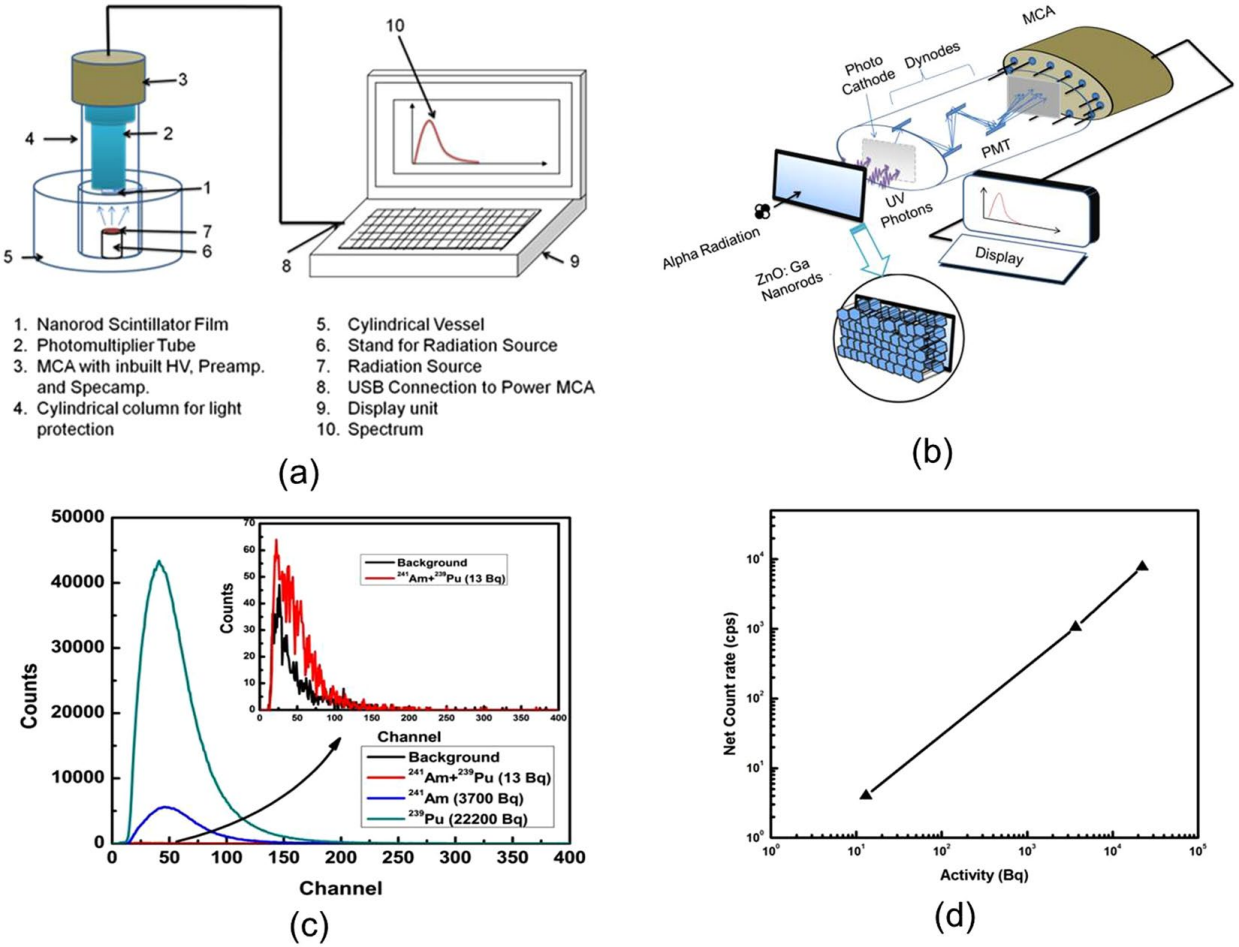
(**a**) The schematic of fabricated alpha radiation detector, (**b**) zoomed view of the detection unit with processes involved in alpha detection, (**c**) recorded pulse height spectrum for different alpha sources, and (**d**) net count rate versus activity for different alpha sources, estimated using counts in (**c**).

The fabricated alpha radiation detector (Fig. 3a) is used to record pulse spectrum for three different sources ^241^Am + ^239^Pu, ^241^Am and ^239^Pu of activities 13 Bq, 3700 Bq, and 22200 Bq, respectively. The distance between the alpha radiation source to detector ~5 mm and counting time ~300 s are kept constant for recording the pulse spectra for all these sources and the recorded spectra are shown in Fig. 3c, with inset showing pulse height spectrum for background and low activity ^241^Am + ^239^Pu alpha source. As can be seen from Fig. 3c that background spectrum intensity is extremely low and the pulse height spectrum is clearly distinguishable for even a low activity alpha source. The corresponding net count rate (calculated by subtracting background count rate (3 cps)) against the activity for different alpha sources is shown in Fig. 3d, and is nearly linear.

### Mechanism of alpha radiation-induced radioluminescence in ZnO:Ga nanorods

**Figure 4:**
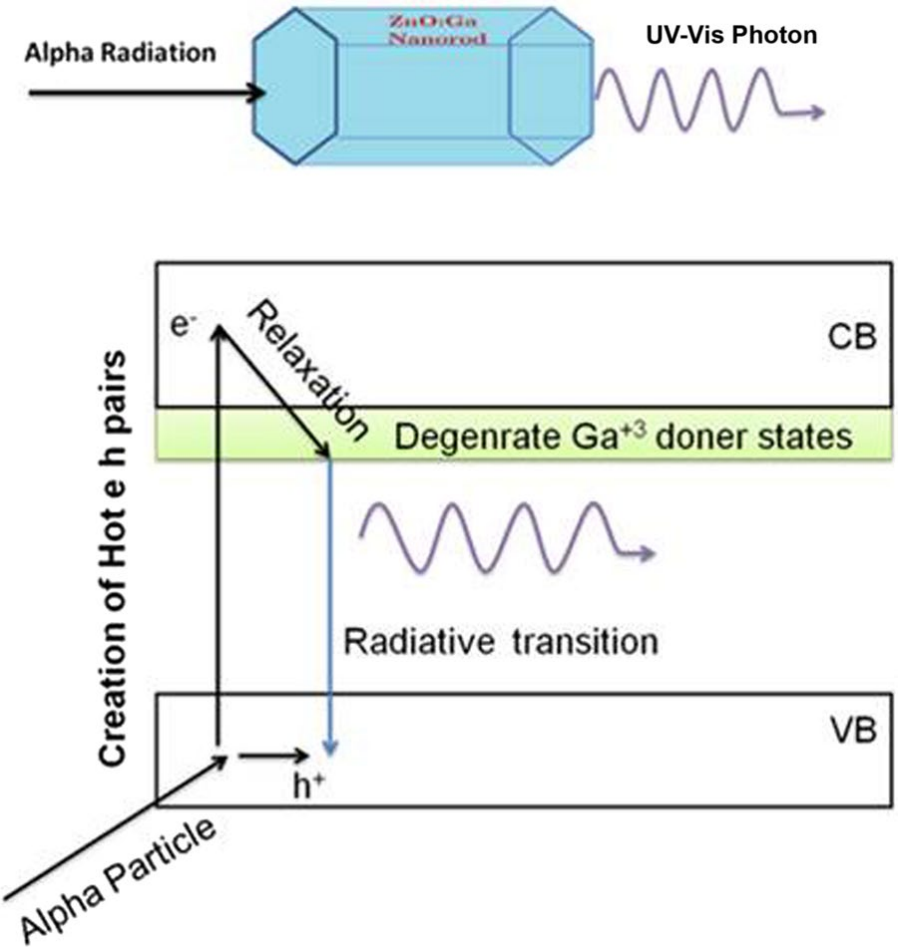
Schematic view of alpha particle induced radio luminescence in ZnO:Ga nanorods with respective electronic transitions.

The doping of Ga^+3^ in ZnO gives rise to the donor defect states below the conduction band^26^ and thus, assisting in filling ZnO trap states. The filling of trap states after Ga doping makes it a fast scintillator^5^. A degenerate doner band is formed which overlap with the edge of conduction band allowing radiative transitions with greater stokes shift^6,27^. Alpha particle carries two positive charges and possesses 4 amu atomic mass. Therefore, it will deposit energy in a short-range creating hot electron-hole pairs. The charged alpha particle interacts trough Coulomb force with the electrons present in the traversing medium. The energy transferred in single collision is 4(*Em*_0_/*m*) where *E* = alpha particle energy, *m*_0_ = electron mass and *m* = particle mass**)**^1^ The energy E of the alpha particle is relatively large (5.5 MeV), there may be a small probability that the high energy electrons after alpha particle interaction may generate secondary electrons. However, the dissipation of energy is relatively faster for alpha particles, and also the energy transferred to the primary electron is only ~1 keV or less in a single collision so the chance of secondary electrons generation will be relatively low or even negligible. The excited electrons will relax thermally to the bottom of the conduction band through a non-radiative process and finally comes to Ga^+3^ donor band, just below the conduction band. This electron subsequently combines with a hole in the valence band, generating a 393 nm UV light signal (Fig. 4).

### Response with the variation of source to detector distance

**Figure 5:**
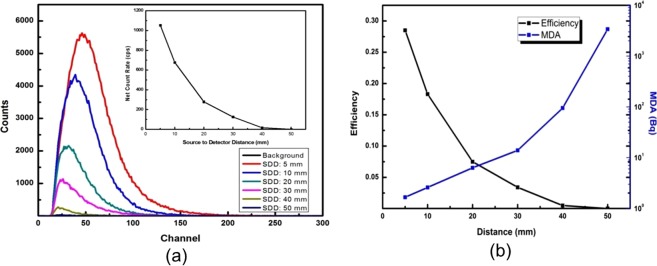
(**a**) Pulse height spectrum of ^241^Am alpha source for different source to detector distances, and (**b**) the calculated absolute detection efficiency and MDA against distance.

The response of the alpha detector is investigated for the different source to detector distances ranging from 5 mm to 50 mm and the recorded pulse height spectra are shown in Fig. 5a with inset showing the corresponding net count rates. The intensity of pulse height counts is decreasing and the spectral peak is shifting towards lower channels as the source is moving away from the detector. This is attributed to the interaction of alpha particles with air molecules prior to reaching the detector, which ended up with fewer alpha particles at the reduced energies reaching the detector. After 40 mm. the pulse height spectrum merges with that of background after 40 mm distance of the source from the detector. This is attributed to the complete loss in energy of the alpha particle as the range of 5.5 MeV alpha particles is about 41 mm in the air. The detection efficiency and minimum detectable activity (MDA) values are calculated (Table 1) and their variation with the source to detector distance is shown in Fig. 5b. As the distance between the source and detector increases, detection efficiency decreases which result in MDA increase. The noticed maximum detection efficiency is ~28.5% and corresponding MDA is 1.7 Bq (Table 1).

**Table 1:** Absolute detection Efficiency calculated at different source to detector distance using ^241^Am Source with counting time 300 sec.

Source to Detector Distance (mm)	Net Source Counts (cps)	Absolute detection efficiency	Minimum detectable Activity (Bq)
5	1054.8	0.285	1.66
10	677.9	0.183	2.59
20	280.4	0.075	6.32
30	126.3	0.034	13.94
40	18.5	0.005	94.80
50	0.5	0.00014	3385.95

Further, the absolute detection efficiency can be maximum 50% in 2π geometry, however, due to various effects such as backscattering, absorption in the mylar window and air, the experimentally measured values are always less than the theoretical value which is around 30%^28^. In the present study, the measured efficiency is 28.5% which is a comparable to this number. Additionally, the alpha radiation detectors are should avoid mylar films in the detector designs to avoid the unintentional absorption, which is commonly used in the conventional scintillator detectors such as ZnS(Ag). The present design of alpha radiation detector offers this advantage as well (Fig. 3), and thus, avoiding unintentional absorption of alpha radiation.

### Repeatability of the alpha detection response

**Figure 6:**
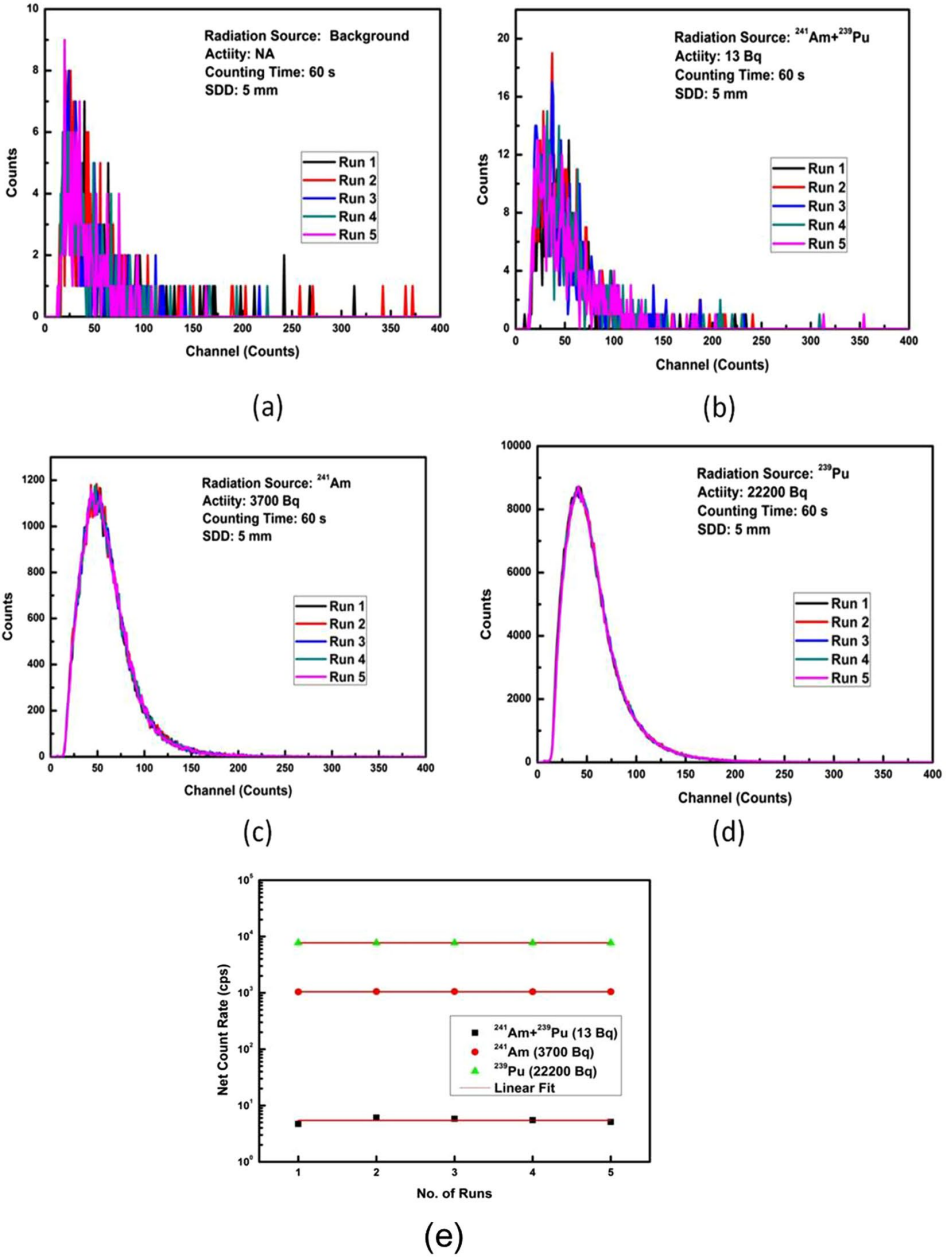
Pulse height spectra of background (**a**), ^241^Am +^239^Pu (**b**), ^241^Am (**c**), and ^239^Pu (**d**) sources recorded five times and (**e**) variation of net count rate against different runs.

The repeatability of the alpha radiation detector response is studied by analyzing the variation in counts for five independent runs/trials. The recorded pulse height spectrums for different runs are shown in Fig. 6(a–d) for background, ^241^Am+^239^Pu, ^241^Am, and ^239^Pu alpha sources. The background is limited to counts less than 10 for each run, Fig. 6a. The relative intensity of alpha radiation for each source is quite high with respect to the background and easily distinguishable. The intensity of counts increases with source radiation intensity. The measurements are highly reproducible and corresponding net count rates are summarized in Table 2. The variation in net count rate due to radiation sources is within ±1%, Fig. 6e, which is in agreement with previous studies carried out on repeatability of ZnO and ZnS(Ag) scintillators^29^

**Table 2:** Count rates of different sources at distance of 5 mm and counting time 60 s.

No. of Runs	Average count rate (cps)			
	Background	^241^Am+^239^Pu (13 Bq)	^241^Am (3700 Bq)	^239^Pu (22200 Bq)
1	2.8	7.5	1044.3	7747.8
2	3.2	8.9	1057.1	7736.6
3	2.7	8.6	1056.9	7735.0
4	2.7	8.3	1049.3	7739.5
5	2.6	7.9	1051.9	7720.3
**Average**	**2.8**	**8.2**	**1051.9**	**7735.8**

### Reproducibility of the alpha detection response

**Figure 7:**
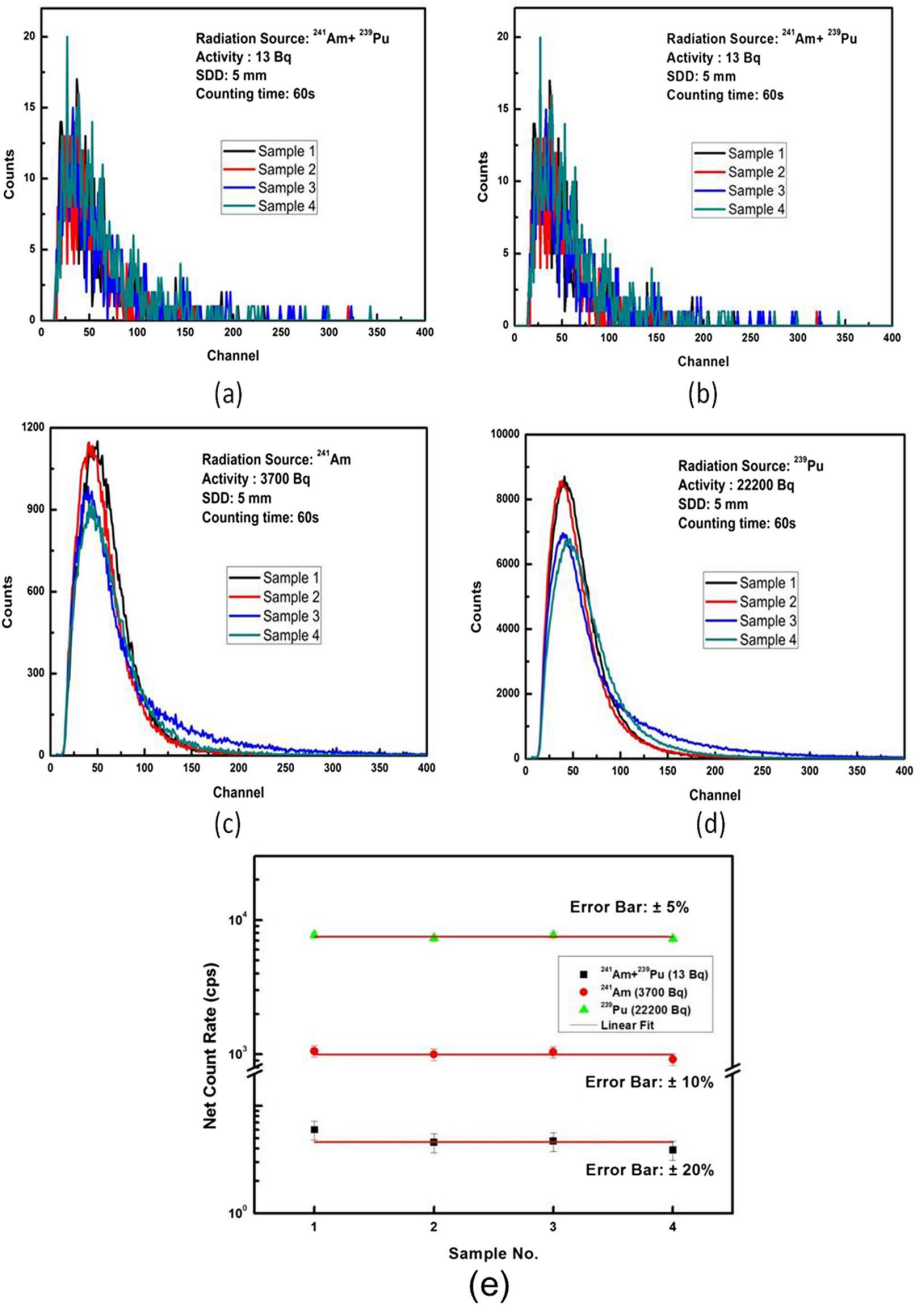
Pulse height spectrum of background (**a**), ^241^Am  +  ^239^Pu (**b**), ^241^Am (**c**) and ^239^Pu (**d**) recorded on four ZnO:Ga nanorod scintillator samples, and (**e**) variations in net count rate for different samples.

Pulse height spectra are recorded on four different ZnO:Ga nanorod scintillator samples, synthesized under identical conditions, for background, ^241^Am+^239^Pu, ^241^Am and ^239^Pu alpha sources. The respective spectra are shown in Fig. 7(a–d) and corresponding net count rates are summarized in Table 3. The detection of alpha radiation is quite robust and respective spectra are reproducible. The error in reproducibility is large for lower intensity, which reduces with increasing the alpha radiation intensity. The variations observed in net counts for these four ZnO:Ga detectors are within ±20% for to ^241^Am+^239^Pu radiation source; ±10% for ^241^Am source and ±5% for ^239^Pu (Fig. 7e). Higher variation is observed for the low activity alpha radiation source and is attributed to statistical fluctuations recorded during the measurements. Increasing the count rate may reduce the variations and thus, lower the respective variations. The radioactive emission of alpha particle is a statistical phenomenon and thus, large variations are expected in case of low activity source. Whereas for large activity source the reproducibility has improved a lot with respect to lower activity sources, due to the improved emission rate statistics.

**Table 3:** Count rate obtained by using four samples of ZnO:Ga detector and different radiation sources.

Sample No.	Av. Counts (cps)			
	Background	^241^Am+^239^Pu (13 Bq)	^241^Am (3700 Bq)	^239^Pu (22200 Bq)
1	2.6	8.6	1056.9	7735.0
2	3.2	7.8	997.9	7359.8
3	4.0	8.7	1042.6	7734.9
4	7.2	11.1	928.6	7272.2

## Conclusion

ZnO:Ga nanorods scintillator is grown successfully using a low-temperature hydrothermal method. Alpha radiation detector is realized using the synthesized nanorod scintillator, showing a promising response to alpha radiation. Detector performance is linear against the activity. Detection efficiency of ~28.5% and minimum detectable activity 1.7 Bq are noticed at 5 mm source to detector distance. Detector showed excellent repeatability within ±1%. The reproducibility of four ZnO:Ga scintillator prepared under identical conditions are found reproducible within ±20% for extremely low-level alpha activity and ±5% for high alpha activity sources. The present study may provide a novel approach to synthesize ZnO:Ga scintillators, showing excellent alpha radiation detection even with low activity.

## Material and Methods

### Synthesis of ZnO:Ga nanorods

(Zn(CH_3_COO)_2_.(H_2_O)_2_) and monoethanolamine are dissolved in isopropyl alcohol (IPA). The solution is stirred vigorously and kept for aging for 24 hrs. A seed layer is synthesized using the spin coating of the aged solution on 1” × 1” ultrasonically cleaned glass substrate and preheated at 300 °C. The process was repeated until the desired thickness of the seed layer is achieved. The substrate is heated at 450 °C to achieve uniform crystalline seed layer. To get the doped nanorods, the dopant precursor gallium nitrate hydrate is dissolved in DI water with (Zn(CH_3_COO)_2_.(H_2_O)_2_) and hexamethylenetetramine. The seed layer coated substrate is placed in the solution with the seeded surface facing down in a substrate holder. The solution is heated at 93 °C for 6 hours in an electric oven. The substrate is allowed to cool down at room temperature and then washed with DI water several times to remove any residual impurities. The substrate is finally heated at 450 °C for 4 hours in air to facilitate highly c-axis oriented ZnO:Ga nanorods.

### Alpha radiation detector

ZnO:Ga nanorods grown on 1″ × 1″ FTO glass substrate is coupled to a photomultiplier tube (Make: Electron Tube, model:9256 KA) with spectral response 300 nm to 650 nm using silicon grease. PMT tube is wrapped using black tape to prevent the interference from external light. 14 Pin PMT base is connected to a commercially available 1 K MCA with an inbuilt voltage divider for PMT, preamplifier and spectroscopy amplifier. MCA is then connected to the control and display system. PMT high voltage and gain can be controlled with spectrum acquisition and analysis software. A long cylindrical column having diameter higher than that of PMT is attached with MCA. Another end of the column is inserted in a cylindrical vessel which acts as the detection and measurement chamber and also as the base for the stability of the unit. The stand of variable sizes can be placed in the chamber for mounting the alpha source. Alpha particles emitted from radiation source interact with ZnO:Ga nanorods (Fig. 3a). UV photons are generated during the interaction and converted into an electrical pulse by the photomultiplier tube. These pulses are analyzed by MCA and displayed as pulse height spectra on control and display system screen as shown in the enlarged view of the detection unit (Fig. 3b).

### Material characterization

Crystallographic information about Ga doped ZnO nanorods is investigated using a ruker X-ray diffractometer (XRD), equipped with Cu K_α_ X-ray source, operating at 40 kV in θ–2θ scan mode with 2θ ranging from 30 to 80. The band gap is measured in the range of 250–700 nm using Cary 4000 UV-Vis Spectrophotometer, (Agilent make). The morphologies of synthesized ZnO:Ga nanorods are investigated using ZEISS make Scanning Electron Microscope. PL measurements are carried out using JASCO 6000 Spectrofluorometer.

### Performance evaluation

The performance of the alpha radiation detector is evaluated by using the different alpha source such as ^241^Am + ^239^Pu, ^241^Am and ^239^Pu of activities 13 Bq, 3700 Bq and 22200 Bq, respectively. Pulse height spectrum of these sources is recorded for a duration of ~‘ 300 s by keeping source at a distance of 5 mm from the detector. The detector response with the variation of a source to detector distance is also studied by varying source to detector distance in the range of 5–50 mm. The repeatability of the detector performance is evaluated by acquiring the spectrum and counts several times with one ZnO:Ga nanorod scintillator and reproducibility of performance is evaluated on four ZnO:Ga nanorod scintillators prepared under identical synthesis conditions by analyzing the pulse height spectra and net counts variation. Detection Efficiency ‘*ε*’ and MDA are calculated using the following formulas;

$$\varepsilon =\frac{{N}_{s}-{N}_{B}}{A}\,and,MDA=\frac{2.71+4.65\sqrt{{N}_{B}\ast t}}{\varepsilon \ast t}Bq$$

Where, N_s_ is counts in cps with the source of activity A, N_B_ background counts in cps, t is counting time.
