# Author Correction: Efficient Alpha Radiation Detector using Low Temperature Hydrothermally Grown ZnO:Ga Nanorod Scintillator

**DOI:** 10.1038/s41598-020-60513-5

**Published:** 2020-02-19

**Authors:** R. M. Sahani, Chandni Kumari, Arun Pandya, Ambesh Dixit

**Affiliations:** 1 Nuclear Radiation Management and Application Division, Defence Laboratory (DRDO), Jodhpur, 342011 India; 2 Department of Physics, Indian Institute of Technology Jodhpur, Jodhpur, 342037 India

Correction to: *Scientific Reports* 10.1038/s41598-019-47732-1, published online 06 August 2019

This Article contains an error in Figure 7, in which Panel A was a duplication of Panel B. The correct Figure 7 appears below as Figure 1.

**Figure 1:**
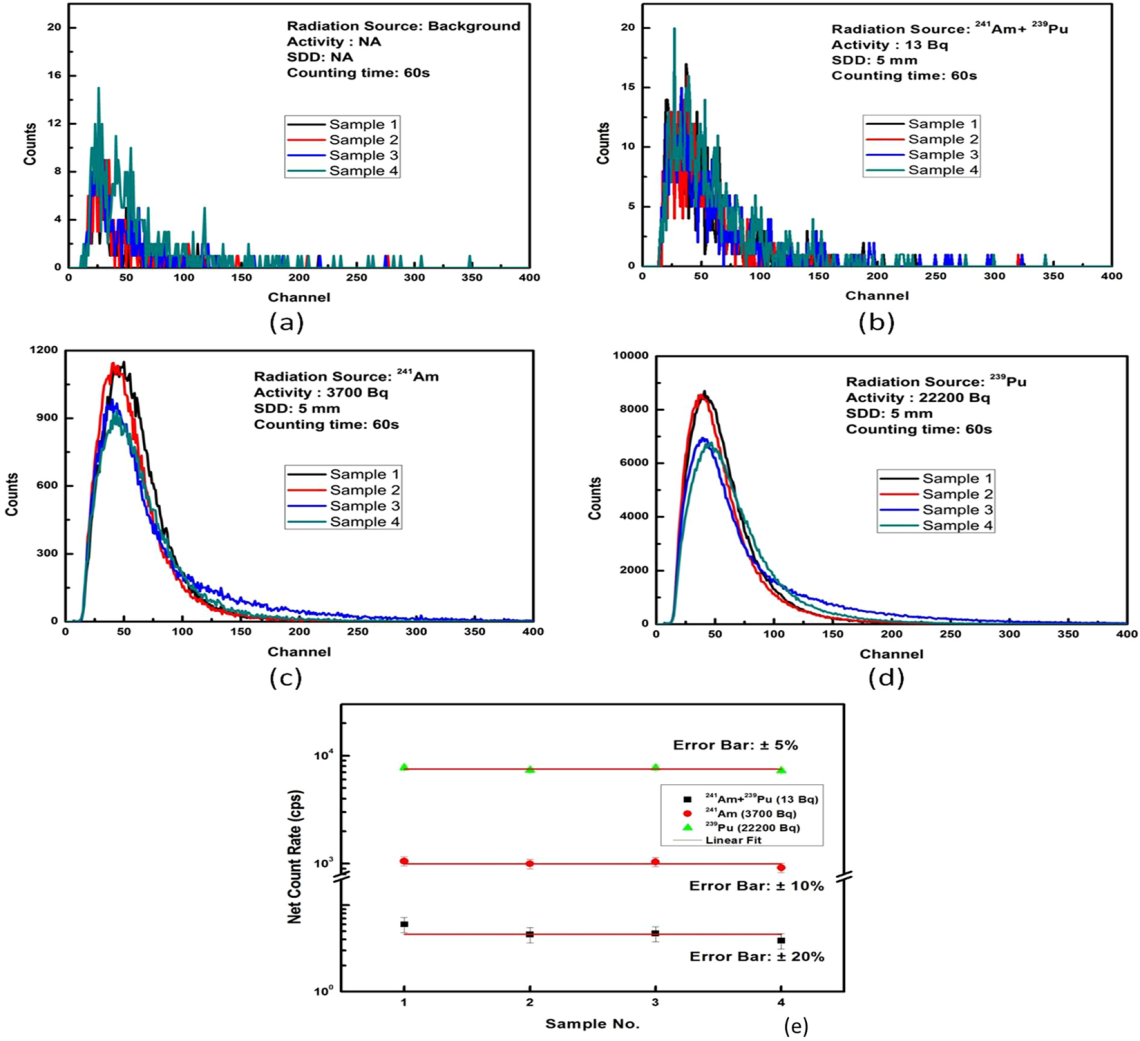
.

Additionally, this Article contains typographical errors in the Introduction:

“Alpha radiation, one form of Ionizing radiation, is a stream of doubly charged particles and is emitted from radioactive decay of a heavy nucleus, D-T or D-D reactions^1^.”

should read:

“Alpha radiation, one form of Ionizing radiation, is a stream of doubly charged particles and is emitted from radioactive decay of a heavy nucleus and D-T reaction^1^.”

“In a neutron generator utilizing D-D to D-T reaction for emission of monoenergetic neutrons for nuclear material interrogation purpose, monitoring of alpha radiation generated in opposite to neutrons is used for associated particle imaging^2^.”

should read:

In a neutron generator utilizing D-T reaction for emission of monoenergetic neutrons for nuclear material interrogation purpose, monitoring of alpha radiation generated in opposite to neutrons is used for associated particle imaging^2^.”

